# Determining the Prevalence of Child Maltreatment Among Young Adults in Saudi Arabia Using ISPCAN Child Abuse Screening Tool

**DOI:** 10.7759/cureus.38531

**Published:** 2023-05-04

**Authors:** Sarah S Aldharman, Lina S Alrasheed, Wed S Alotaibi, Asma M Alqahtani, Reem M Bajrai, Hassan Saleheen, Maha A Almuneef

**Affiliations:** 1 Medicine, King Saud Bin Abdulaziz University for Health Sciences, Riyadh, SAU; 2 Epidemiology and Public Health, King Abdullah International Medical Research Center (KAIMRC), Riyadh, SAU; 3 Pediatrics, King Abdullah Specialized Children's Hospital, Riyadh, SAU

**Keywords:** neglect, saudi arabia, prevalence, abuse, child maltreatment

## Abstract

Background

Child maltreatment is an important public health issue, thus determining its prevalence is critical to recognize the extent of the problem and mandate efforts to combat child abuse. We aimed to investigate child maltreatment prevalence among special populations of young adults in Riyadh, Saudi Arabia.

Methods

We used the retrospective version of the International Society for Prevention of Child Abuse and Neglect (ISPCAN) Child Abuse Screening Tool (ICAST-R). The survey included Saudi students of both genders aged between 18 to 24 years old and attending King Saud bin Abdulaziz University for Health Sciences (KSAU-HS). The questionnaire was provided electronically using Survey Monkey (Momentive Global Inc., San Mateo, CA, USA).

Results

A total of 713 students completed all sections of the questionnaire. The prevalence of any type of child maltreatment was estimated to be 42%. Physical abuse was the most prevalent (51.1%), followed by emotional abuse (49.9%), lack of protection and safety (38%), and sexual abuse (29.6%). The most common form of physical abuse was being hit or punched at 77.5% followed by 'beaten very hard with an object' at 58.8% while touching was the most common form of sexual abuse at 68.7%, and only 13.7% encountered penetrating form of sexual abuse. In comparison to female victims, male victims were more likely to be physically abused (odds ratio (OR)=1.5; confidence interval (CI)=1.1-2.0). Participants who lived with a single parent were more likely to be victims of a lack of protection and safety than those who lived with both parents (OR=1.9; CI=1.0-3.7). Most participants reported the abuse to occur after the age of nine years, and the perpetrator was the parents in 17.5% of cases.

Conclusion

Our findings demonstrated a high prevalence of child maltreatment among the young adult population in Saudi Arabia. It is vital to obtain more information on the prevalence and risk factors of child maltreatment in various populations and regions of Saudi Arabia to raise awareness and improve services for the victims of abuse.

## Introduction

Childhood plays a significant role in shaping a person's personality and behavior. Therefore, anything that disturbs the normal development of a child, such as maltreatment, is considered a brutal act [1]. Children need to grow in a healthy environment where they are loved and treated properly so they can develop into normal healthy individuals. Child maltreatment is defined as “any act(s) of commission or omission that results in harm, the potential for harm, or threat of harm to a child, or the failure to provide for a child’s needs or to protect a child from harm or potential harm" [2]. Acts of commission (child abuse) include intentional words or actions, but the harm to a child might or might not be the intended consequence. It can be in any form including physical, sexual, or emotional abuse. On the other hand, the act of omission (child neglect) is defined as “the failure to provide for a child’s basic physical, emotional, or educational needs or to protect a child from harm or potential harm” [2]. A caregiver's failure to offer health care, physical, emotional, social, or educational needs is an example of child neglect. Therefore, it is challenging to accurately measure the extent of this public health problem in a society because it occurs behind the scenes and thereafter, unseen. Child maltreatment has been identified as a major social health issue around the world [1]. Many efforts have been made to estimate the prevalence of child abuse in several countries [1]. The International Society for the Prevention of Child Abuse and Neglect (ISPCAN) and the United Nations International Children’s Emergency Fund (UNICEF) cooperatively have constructed ISPCAN Child Abuse Screening Tools (ICASTs), which are international tools used to enable information-gathering about the prevalence of child maltreatment, determine the scope and extent of the problem, and investigate the various types of child maltreatment across the world [3]. Using this kind of instrument allows for an accurate estimation of prevalence and a proper comparison between countries [4]. The ICAST questionnaires were developed in three different versions, namely ICAST-P for parents, ICAST-CH for children, and ICAST-retrospective (R) for young adults [3].

The World Health Organization fact sheet 2020 report shows that nearly three in four children aged two to four years are exposed to physical and/or psychological abuse regularly by their parents or caretakers [5]. Also, one in every five women and one in every 13 men report having been sexually abused as a child between the age of zero and 17. In Saudi Arabia different data management tools and services were able to collect data on the incidence of child maltreatment; however, statistics on the prevalence of the problem are lacking. The National Family Safety Registry (NFSR) collected data from all hospital-based child protection centers (H-CPC) in all 13 provinces that give an estimate of the incidence of the problem [6]. The H-CPC based in King Abdulaziz Medical City in Riyadh, Saudi Arabia reported 220 cases of child abuse and neglect (CAN) between 2009 and 2013. Physical abuse was the highest (42%) followed by neglect (37%), sexual abuse (13%), and then emotional abuse [7]. The Saudi Child Helpline (116-111) also records cases of child abuse reported directly from the community. In 2020, over 8000 reports of child abuse were conveyed to the helpline [8]. These incident reports can identify the percentages of different forms of child abuse, risk factors, and outcomes; however, they will not ascertain the magnitude of the problem at all levels of society. Therefore, it is crucial to get an assessment of the prevalence of this problem by piloting a population survey to know the estimated number of maltreated children to initiate protection services and prevention strategies based on the evidence. 

Maltreatment can affect a child in many aspects including brain function, structure, and connectivity. It causes pathways and sensory system alteration which convey unfortunate experiences [9]. Impairment in academic performance is another effect of child maltreatment [10]. Furthermore, other studies reveal that child maltreatment creates a significant burden in society with not only medical costs but also legal expenses and low productivity which affects the social and economic growth of the country [1]. The ICAST questionnaire, which has been used in many developing and developed nations, estimates the prevalence of child maltreatment in a particular country using either the ICAST-CH, ICAST-R, or ICAST-P. Using different ICAST instruments, child abuse prevalence may be addressed internationally in a standardized, cross-cultural manner. In a study on 192 students in the first year of medical school at a Turkish university, 56.8% of females and 43.2% of males were assessed using ICAST-R. Physical abuse was reported by 14.6% of participants, emotional abuse by 32.3 %, and sexual abuse by 8.9% [11]. Another study included 6682 students from 18 schools in Kerala, India, who were surveyed using ICAST-CH to estimate the prevalence of child abuse [12]. The prevalence was high, and it showed that the most common type of abuse was emotional abuse. In that study, several factors were noted to raise the likelihood of abuse including low socioeconomic level, alcohol, and drug consumption by family members at home, and male gender [12]. Recent research was conducted using ICAST-R to investigate the prevalence of child maltreatment in Ecuador [13]. A total of 3133 students participated from the seven largest universities in Ecuador. The data revealed that 69.6 % had experienced child maltreatment. Physical abuse was stated by 47.6% of respondents, with the most prevalent form being beaten by parents. Around 53% reported emotional maltreatment often by insults by same-sex peers and parents, and 15.5 % reported being sexually abused. Females were more likely to be sexually abused, whereas males were more likely to be physically abused [13]. In Saudi Arabia, the ICAST-CH was used to determine the prevalence of child maltreatment in the region and was administered to school-aged children between the age of 15 to 19 years [14]. This cross-sectional study was carried out in the country's five major regions (eastern, central, western, northern, and southern regions) where 16,939 students attending intermediate and high school were enrolled. Emotional abuse was reported to be the most prevalent type, accounting for 65% of all cases, while sexual abuse was the least common, accounting for 10%. The study reveals that girls are more prone to witnessing violence, emotional abuse, and neglect, while boys reported more physical and sexual abuse [14]. To validate the findings and reliably evaluate the prevalence of child abuse in Saudi Arabia, it may be necessary to conduct more studies using different forms of the ICAST questionnaire.

Unfortunately, the scarcity of studies in Saudi Arabia on this ongoing problem using a standardized tool such as ICAST-R would not allow us to compare our results to other countries. This is an important subject that deserves more attention because it involves children who represent the future and the long-term effects of abuse on adulthood. Therefore, the purpose of this study was to estimate the prevalence of child maltreatment among college students at King Saud bin Abdulaziz University for Health Sciences, Riyadh, using ICAST-R. Also, it aimed to identify the various types of child maltreatment and the associated risk factors. Consequently, such data will help recognize the extent of the problem and raise awareness of child maltreatment in the community and among government organizations to make more efforts in combating child abuse in the country. This data also aids in identifying trends in prevalence and assessing newly implemented protection programs available in the country such as legislation, shelters, social services, parenting, and awareness programs. 

## Materials and methods

Study design and settings

A cross-sectional questionnaire-based study was conducted on college students at KSAU-HS. It is a government university specializing in health sciences and was established in 2005. The university campus in Riyadh has seven colleges: College of Medicine, College of Dentistry, College of Pharmacy, College of Applied Medical Sciences, College of Nursing, College of Public Health and Health Informatics, and College of Sciences and Health Professions. The academic courses at the university are linked to the training programs in King Abdulaziz Medical City, Ministry of National Guard - Health Affairs, where the student has the opportunity for clinical training under the supervision of senior consultants and specialists in different diagnostic and therapeutic departments.

Study participants

The inclusion criteria of the study were all undergraduate students at KSAU-HS (approximately 3790 students). The sample included both genders studying in the pre-clinical and clinical phases. The participants were between 18 to 24 years old and agreed to participate and complete the questionnaire. Non-probability convenience sampling technique was used. Using the Raosoft sample size calculator (Raosoft Inc., Seattle, WA, USA) a representative sample size of 349 was arrived at while keeping the margin of error at 5% and the confidence level at 95%.

Data collection process

**Figure 1:**
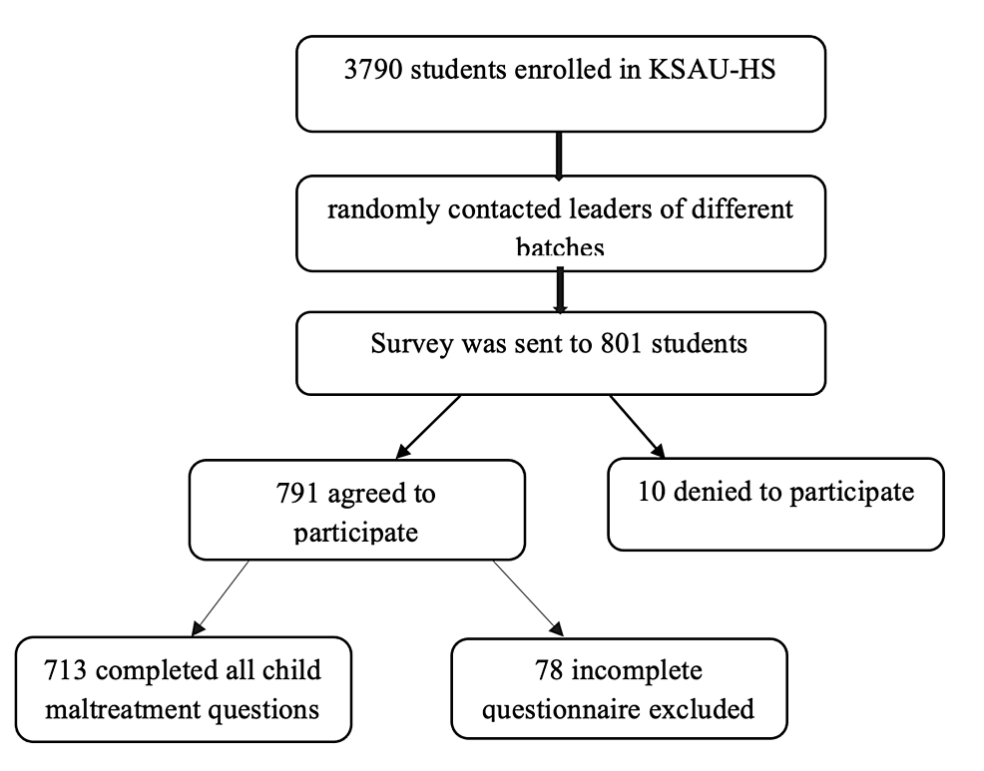
Consort chart of the data collection process KSAU-HS: King Saud bin Abdulaziz University for Health Sciences

The ICAST-R questionnaire developed by ISPCAN and UNICEF was applied to collect information from participants [3]. The English language questionnaire was used for our target population. The ICAST-R is a valid and reliable questionnaire used in previous studies. Delphi study results revealed that ICAST-R performed well cross-culturally in seven different countries and languages [15]. It is a self-administered questionnaire for the age group 18 to 24 years and retrospectively measures experiences of physical, emotional, and sexual abuse and neglect during childhood. The questionnaire ensured confidentiality for the participants and did not require the participants' names. It includes 36 items about lifetime exposure before age 18 to physical and emotional events and sexual abuse. The first eight questions inquire about the participants’ socio-demographic features such as gender, age, level of education, employment, and accommodation status. However, we excluded the questions about the level of education and employment status (does he/she currently work or not) because we targeted university students who are not working. Also, we added a question asking about the type of college the participants are currently studying in. Questions 9 to 16 are about neglect experiences and their details. Questions 17 to 23 query physical abuse experiences and their details, 24 to 30 ask about emotional abuse and its details, and 31 to 36 inquire about sexual abuse and its details. There are three choices to all questions: "yes", "no", and "cannot remember". Each question was followed by supplemental questions asking about frequency, duration of abuse, and the perpetrators. Leaders of different batches in the university were contacted randomly and requested to send the questionnaire to their groups. The study was approved by the Institutional Review Board of King Abdullah International Medical Research Center, Ministry of National Guard - Health Affairs, Riyadh, Kingdom of Saudi Arabia (approval number: SP20/325/R). The questionnaire was provided electronically using Survey Monkey (Momentive Global Inc., San Mateo, CA, USA). Before filling out the questionnaire, the informed consent form was provided to the participants. After filling out the questionnaire, they were checked for completeness. Any questionnaire that was not fully completed was excluded (see the consort chart in Figure 1). 

Data analysis

Data were entered in Microsoft Excel (Microsoft Corp., Redmond, WA, USA) and then transferred to SPSS software version 27.0 (IBM Corp., Armonk, NY, USA). The chi-square test was used to compare categorical variables such as gender, level of education, and accommodation status with the outcome, and presence or absence of abuse. Logistic regression analysis was conducted by calculating odds ratios (ORs) and corresponding 95% confidence intervals (CIs) for each of the potential explanatory variables about the outcome. A p-value <0.05 was considered significant. 

## Results

The total number of students enrolled in KSAU-HS in 2021 was 3790. The questionnaire was sent to the university students through different batch leaders. The number of students who clicked on the link and downloaded the survey was 801 (21.6%) of which, 713 agreed to participate and complete the questionnaire with an overall response rate of 89%. 

The socio-demographic characteristics of the participants are shown in Table 1. A little over half (58.8%) were 21 to 23 years old, followed by the age group 18 to 20 years (31.4%), and ≥24 years (9.8%). Nearly half (48%) were male, while 52% were female. When participants were growing up, most of them (93.1%) lived in a big city while 6.9% lived in a village or small town. The majority of participants lived in a nuclear family setting with mothers, fathers, and siblings (93%, 88%, and 91%, respectively) while few lived with extended family members that included grandmothers, grandfathers, and others (11.7%, 6.4%, 3%, respectively). The participants were distributed evenly in all seven colleges based on the total number of students in each college with the majority being from the College of Medicine (35.5%). 

**Table 1:** Socio-demographic characteristics of the participants (N=713)

Variables	Number (%)
Age category	
18-20 years	224 (31.4)
21-23 years	419 (58.8)
24 years	70 (9.8)
Gender	
Male	342 (48.0)
Female	371 (52.0)
Growing up in	
A big city	664 (93.1)
A small town/city/small village	49 (6.9)
Living in the same house with	
Father	628 (88.1)
Mother	666 (93.4)
Grandfather	46 (6.5)
Grandmother	84 (11.8)
Stepparent	22 (3.1)
Siblings	648 (90.9)
Attended college	
College of Medicine	253 (35.5)
College of Applied Medical Science	174 (24.4)
College of Nursing	80 (11.2)
College of Science and Health Profession	75 (10.5)
College of Pharmacy	64 (9.0)
College of Dentistry	45 (6.3)
College of Public Health and Health Informatics	9 (1.3)

**Figure 2:**
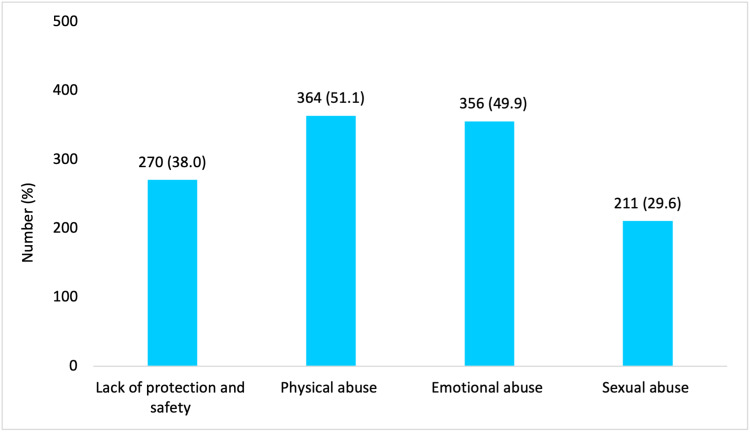
Prevalence of different forms of child abuse among participants (N=713)

**Figure 3:**
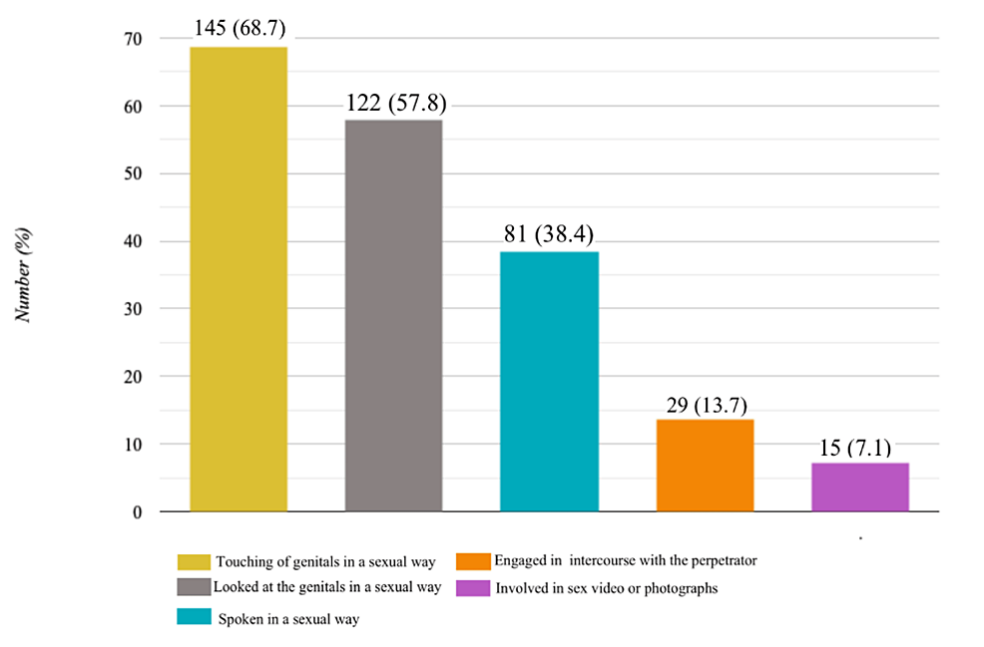
Prevalence of different types of sexual abuse among participants reporting acts of sexual assault (N=211)

Physical abuse was the most common form of abuse accounted for by 364 students (51.1%), followed by emotional abuse by 356 (49.9%), lack of protection and safety by 270 (38.0%), and sexual abuse by 211 (29.6%) (Figure 2). The prevalence of being hit or punched very hard was the most common form of physical abuse in 282 students (77.5%), followed by being beaten very hard with an object in 212 (58.5%), kicked very hard was reported by 140 (38.5%), being shaken very hard was reported by 52 (14.3%), and being stabbed or cut with a knife/sharp object was reported by 38 (10.4%). More than two-thirds i.e., 145 (68.7%) participants reported sexual abuse and reported being touched in their private parts or being made to touch the perpetrator sexually; followed by 122 (57.8%) who were made to look at the perpetrator's private parts or the perpetrator looked at theirs, 81 (38.4%) were spoken to sexually or had sexual things written about them, 29 (13.7% ) were coerced into sexual intercourse with the perpetrator, and only 15 (7.1%) reported that they were forced to participate in a sex video or had photographs taken of them (Figure 3). Almost all of the sexual abuse cases reported a ‘non-penetrating’ act (208 (98.6%)), while 29 (13.7%) reported ‘penetrating’ sexual abuse, and 15 (7.1%) had non-contact sexual abuse. Only 13 (6%) sexual abuse victims reported the sexual assault to an adult. There were 27 different reports to different people issued by the victims, of which female victims account for only eight (30%) of these reports, while male victims account for 19 (70%). Male victims reported it to parents and teachers while female victims reported it to parents and peers, and none of the 27 reports went to the police, with only one report each going to a social worker and medical doctor.

A comparison of the age at which children experienced acts of abuse is shown in Table 2. Most participants reported the abuse to occur at an age over nine years old compared to children less than nine years old (p<0.05). Only 2% reported the abuse to occur at age less than five years, 16% at age five to nine, 45% at age 10 to 13, and 35% at over 14 years old. 

**Table 2:** Comparison of age at which a young person experienced an act of abuse

Type of abuse	<5 years	5 to 9 years	10 to 13 years	>14 years
	# (%)	# (%)	# (%)	# (%)
Physical	4 (1%)	53 (15%)	190 (54%)	107 (30%)
Sexual	1 (0.5%)	53 (26%)	87 (42%)	65 (32%)
Emotional	4 (1%)	22 (6%)	137 (40%)	182 (53%)
Neglect	14 (7%)	56 (26%)	104 (48%)	43 (20%)

Parents (17.5%) were the highest reported perpetrators, followed by siblings (10.4%), peers (8.9%), relatives (8.1%), teachers (6.1%), and strangers (5.9%). Parents, siblings, and peers were the most frequently reported perpetrators of physical abuse; whereas peers, relatives, and strangers were the most frequently reported perpetrators in cases of sexual abuse.

The relationship between the demographic characteristics of the participants and the types of abuse is shown in Table 3. Significant differences were found for types of abuse concerning gender and living arrangement. Males were more likely to experience physical abuse (p<0.01). Participants who lived with a single parent were more likely to experience a lack of protection and safety (p<0.05).

**Table 3:** Relationship between the types of abuse and socio-demographics among participants (N=713) †: p<0.05; ††: p<0.01

Types of child abuse	Gender^††^		When you were growing up, where did you live for most of that time?		Who else lives in the same house as you?^†^		
	Male n=342 (%)	Female n=371 (%)	A big city n=664 (%)	A small town n=49 (%)	Nuclear family (father, mother, and sibling) n=550(%)	Extended family (father, mother, sibling, and grandparents) n=66(%)	Other (e.g., living with a single parent) n=97(%)
Lack of protection and safety	122(35.7)	149(40.2)	251 (37.8)	20 (40.8)	201 (36.5)	22 (33.3)	48 (49.5)
Physical abuse	193(56.4)	171(46.1)	335 (50.5)	29 (59.2)	272 (49.5)	40 (60.6)	52 (53.6)
Emotional abuse	169(49.4)	187(50.4)	330 (49.7)	26 (53.1)	280 (50.9)	30 (45.5)	46 (47.4)
Sexual abuse	110(32.2)	101(27.2)	193 (29.1)	18 (36.7)	156 (28.4)	25 (37.9)	30 (30.9)

Table 4 shows the OR for factors associated with lack of protection and safety, physical abuse, emotional abuse, and sexual abuse. Male victims were 1.5 times more likely to be physically abused than female victims. Participants who lived with a single parent were 1.9 times more likely to be victims of a lack of protection and safety as compared to those who lived with both parents. Physical and sexual abuse is more likely to occur in children living in an urban area compared to rural areas (OR 1.3; CI .7-2.5).

**Table 4:** Factors associated with different types of child maltreatment among participants in Saudi Arabia †: Reference is female gender, ‡: Reference is 'grew up in a small city/town', ¥: Reference is 'lived with extended family', OR: Odds ratio, CI: Confidence interval

	Lack of protection and safety		Physical abuse		Emotional abuse		Sexual abuse	
	OR	95% CI	OR	95% CI	OR	95% CI	OR	95% CI
Victim being male^†^	.8	.6-1.1	1.5	1.1-2.0	.9	.7-1.2	1.2	.9-1.7
Grew up in a big city^‡^	1.1	.6-2.0	1.3	.7-2.5	1.1	.6-2.0	1.3	.7-2.5
Lived with single parent^¥^	1.9	1.0-3.7	.7	.3-1.4	1.0	.5-2.0	.7	.3-1.4

## Discussion

This study is a cross-sectional investigation targeting college students in the Riyadh region of Saudi Arabia via ICAST-R. The study measured the frequency of different types of abuse in young adults by asking them about their childhood experiences of abuse. It is the first study in Saudi Arabia to apply the international instrument of ICAST-R which allows us to compare our maltreatment prevalence, types, and risk factors to other countries worldwide. According to the UN Secretary General’s study on violence against children, most children who were subjected to abuse and neglect experienced it at home by their parents [16]. Therefore, it is important to study violence against children at home in different countries to identify the extent of the problem, its risk factors, and its consequences, to raise awareness and provide services to the victims and their families.

Research on child maltreatment in the Arab world is scarce, and the countries in the Middle East and North Africa (MENA) region do not have adequate population-based data on its prevalence or the associated risk factors. Recent studies have revealed that CAN is prevalent in Arab nations yet underreported [17,18]. In the Kingdom of Saudi Arabia, the first study to address the prevalence of child maltreatment was done using ICAST-CH on a sample of 16,939 school-age children between 15 to 19 years old [14]. The prevalence of various types of abuse was highest for psychological abuse at 65%, followed by physical abuse at 50%, and the lowest was sexual abuse at 10% [14]. These results are very similar to what we find in the current study for psychological (49.9%) and physical abuse (51.1%), indicating the consistency of the various ICAST surveys in detecting the accurate prevalence of CAN in various populations. However, the rate of sexual abuse was higher in our study (29.6%) compared to the ICAST-CH study (10%) by Al-Eissa et al. [14], this difference may be related to the age of the participant where the older the victims the higher the chance of disclosing the abusive incident. Different developing countries have used ICAST-R to determine the prevalence of CAN and enable them to compare their results to neighboring countries [13,19,20]. Turkey, using ICAST-R, found that 51.4% of first-year college students were exposed to at least one type of abuse with a higher prevalence rate among the male gender [19]. Ecuador reported that 69.6% of participants had been subjected to child maltreatment, with 47.6% experiencing physical abuse [13]. On the other hand, Qatar used the same ICAST-R on 697 young adults through a random household survey and found the rate of different types of child abuse were 22.1%, 15.6%, and 2.5% for physical, psychological, and sexual abuse, respectively [20]. In South Korea, they performed a study on 539 young persons from different universities and found the rate of physical, emotional, and sexual abuse at 42.2%, 36.3%, and 24,.3%, respectively [21]. The different prevalence rates in different countries using the same tool allow the proper comparison of prevalence and allow each country to follow the trend of their prevalence over time [15]. 

Our data implied that boys and girls are at different risk levels for different forms of child maltreatment, which has been widely debated by child maltreatment experts [22]. This is supported by a study that revealed females report more emotional and sexual abuse than males [23]. In our study, males reported more physical and sexual abuse compared to females; however, a meta-analysis of the prevalence of childhood sexual abuse (CSA) around the world reported that girls have a greater risk of sexual abuse compared to boys [22]. Variations in child protection programs, cultural disparities in how children's rights are interpreted, and differences in the social priority given to boys and girls, as well as differences in parental views and practices, are likely to be linked to gender-specific patterns of child abuse [22]. Many parents think that physical punishment is not considered violence if it is properly administered and used to teach the child a lesson. As reported by a previous study, in 94% of the studies, significant associations were observed between spanking and corporal punishment with subsequent deterioration in child behavior and development, concurrently or later [24]. This is also compatible with the findings of a study that states that children exposed to corporal punishment were about 24% less likely to be developmentally on track than children who were not exposed to corporal punishment [25]. Moreover, there are gender disparities in how parents apply physical punishment, with males receiving more severe versions as they are seen as tougher and therefore are more likely to be physically punished. Also, due to the anticipated masculine duties that might expose them to physical punishment, they may engage in more aggressive or adventurous behaviors. This might explain why our study reported such a high prevalence of physical abuse among males. The lower disclosure of female sexual abuse compared to males in this study and others is noted mainly in data from developing countries compared to Western countries. For instance, according to the CDC, almost one in five women in the United States (19.3%) has been raped at least once in her life, including completed and attempted forced penetration [26]. Another study reported that in high-income Western countries, annually about 4% to 16% of children are physically abused and one in 10 is neglected or psychologically abused. In addition, the study revealed that during childhood, between 5% and 10% of girls and up to 5% of boys are exposed to penetrative sexual abuse, and up to three times this number are exposed to any type of sexual abuse [27]. This disparity may be related to the under-reporting of girls' sexual abuse because it may be regarded as too sensitive for girls and more stigmatizing due to the established concept of virginity related to females only. It is also worth noting that in Saudi Arabia, females are more strictly monitored and less outgoing than males, thereby lowering the likelihood of sexual abuse. Furthermore, gathering more data on Saudi Arabian children's gender-specific vulnerabilities might offer policymakers essential and required knowledge for developing gender-appropriate preventive and intervention measures.

The relationship between the living arrangement of the participants and types of abuse is noted in this study; participants who lived with a single parent were 1.9 times more likely to experience neglect than those who lived with both parents. A previous Saudi study revealed that children who live with a single/step-parent were four times more likely to experience physical abuse [7]. Similarly, the study by Al-Eissa et al. reported that greater rates of abuse/exposure were observed among participants who lived with their mother or father only (versus with both), and even greater rates for all types of abuse when they lived with their biological parent and a step-parent [14]. This may be due to increased responsibility to a single parent, which makes providing for children's needs harder compared to living with both parents. A comparison of the age at which children experience acts of maltreatment showed a significant difference. Older children (>9 years) were more likely to experience all types of maltreatment. In comparison to the study done in Qatar, there was a gender significant difference as it showed that males were more likely to experience physical abuse than females in those who are above nine years old (10 to 13 years: M=14.4%, F=6.5%, p<0.001; 14 to 17 years: M=8%, F=3.2%, p<0.006) [20]. Moreover, another study conducted in Pakistan showed that all types of abuse were higher in children above nine years old [25]. This finding could be due to recall bias, in which participants do not remember acts of maltreatment before the age of nine. In our study, the most common perpetrators were parents, which was the same in the study conducted in Pakistan (17.5% vs 20%). This could be due to poor parenting techniques and a lack of awareness of alternative discipline methods [25]. 

Strengths & limitations

Given the difficulties in revealing abuse experiences, an international retrospective tool such as the ICAST-R might be a valuable instrument in child maltreatment-related research, especially when the vast majority of child maltreatment occurrences go unreported, and information received from parents or the system does not present a complete picture [28]. Another area of strength in our study is that the questionnaire achieved a high response rate. Additionally, retrospective methods help give reliable data on what participants regard to be key life events [29,30]. However, we must consider the possibility of recall bias when using a retrospective method. Another limitation is that the study was conducted at a single governmental university, which makes it less representative of all students in the central provenance of Riyadh, and the findings reflect only one center with a limited number of participants and might not be generalizable to other settings. Another limitation is that this study was conducted in a university that included people who had the chance to have a higher educational level. Therefore, those who did not have the opportunity to attend university and reach a high education level may also be more or less exposed to maltreatment compared to those who are enrolled in a university. Although data collection was administered anonymously, some students may have been reluctant to participate in the study due to fear of exposing their state of abuse. Also, as the questionnaire involves questions exploring abuse and neglect, it may have been hard for the participants to answer such questions about their private life, and this might have led to underreporting and underestimation of actual abuse. This study included participants in one region; therefore, further multi-region studies are recommended to understand the underlying risk factors and prevalence with a larger and more diverse sample that is representative of Saudi Arabia.

## Conclusions

In the Kingdom of Saudi Arabia, there is a lot of development in the efforts to prevent and protect children against maltreatment. The Saudi child helpline, hospital-based child protection teams (H-CPTs), and child protection laws and policies are examples of these efforts. However, this is an ongoing problem as our findings reveal that children are highly susceptible to maltreatment. Physical abuse was the most common form indicating the frequency of physical discipline in society. Older children (>9 years), males, and those living in a single-parent household were more likely to experience child maltreatment. These findings help emphasize the importance of designing interventions and preventative programs tailored to each country's specific vulnerabilities among children and their families. Our research also highlights the significance of obtaining local data on child maltreatment using standardized reliable tools such as the ICAST to enable further national and worldwide comparisons.
